# Hessian Fly-Associated Bacteria: Transmission, Essentiality, and Composition

**DOI:** 10.1371/journal.pone.0023170

**Published:** 2011-08-16

**Authors:** Raman Bansal, Scot Hulbert, Brandi Schemerhorn, John C. Reese, R. Jeff Whitworth, Jeffrey J. Stuart, Ming-Shun Chen

**Affiliations:** 1 Department of Entomology, Kansas State University, Manhattan, Kansas, United States of America; 2 Department of Plant Pathology, Washington State University, Pullman, Washington, United States of America; 3 United States Department of Agriculture-Agricultural Research Service and Department of Entomology, Purdue University, West Lafayette, Indiana, United States of America; 4 Department of Entomology, Purdue University, West Lafayette, Indiana, United States of America; 5 Hard Winter Wheat Genetics Research Unit, United States Department of Agriculture-Agricultural Research Service, Kansas State University, Manhattan, Kansas, United States of America; Instituto Butantan, Brazil

## Abstract

Plant-feeding insects have been recently found to use microbes to manipulate host plant physiology and morphology. Gall midges are one of the largest groups of insects that manipulate host plants extensively. Hessian fly (HF, *Mayetiola destructor*) is an important pest of wheat and a model system for studying gall midges. To examine the role of bacteria in parasitism, a systematic analysis of bacteria associated with HF was performed for the first time. Diverse bacteria were found in different developmental HF stages. Fluorescent *in situ* hybridization detected a bacteriocyte-like structure in developing eggs. Bacterial DNA was also detected in eggs by PCR using primers targeted to different bacterial groups. These results indicated that HF hosted different types of bacteria that were maternally transmitted to the next generation. Eliminating bacteria from the insect with antibiotics resulted in high mortality of HF larvae, indicating that symbiotic bacteria are essential for the insect to survive on wheat seedlings. A preliminary survey identified various types of bacteria associated with different HF stages, including the genera *Enterobacter*, *Pantoea*, *Stenotrophomonas*, *Pseudomonas*, *Bacillus*, *Ochrobactrum*, *Acinetobacter*, *Alcaligenes*, *Nitrosomonas*, *Arcanobacterium*, *Microbacterium*, *Paenibacillus*, and *Klebsiella*. Similar bacteria were also found specifically in HF-infested susceptible wheat, suggesting that HF larvae had either transmitted bacteria into plant tissue or brought secondary infection of bacteria to the wheat host. The bacteria associated with wheat seedlings may play an essential role in the wheat-HF interaction.

## Introduction

Higher eukaryotic organisms including insects host diverse beneficial bacteria [1]–[4]. These bacteria can perform a range of functions that benefit their hosts, including synthesis of essential amino acids [5], digestion of food ingredients that are otherwise inaccessible to their hosts [6], [7], and facilitating host reproduction [7], [8] and stress tolerance [2], [9]–[12]. Bacteria and related microbes also play an important role in inter-species interactions [13], [14]. In insect-plant interactions, phytoplasmas, a group of specialized biotrophic bacteria hosted by the leafhopper *Macrosteles quadrilineatus*, secrete effectors into host plants of the insect that alter the phenotypic appearance of the plant to make it more attractive to the leafhopper [15], [16]. Gall midges are also insects that alter plant function, including the formation of nutritive tissue at the feeding site [17], and in many cases, the formation of out-growth galls [18]. It is not known whether bacteria associated with gall midges play any role in nutritive cell and gall formation.

The Hessian fly (HF, *Mayetiola destructor*) is a gall midge and a serious pest of wheat [19], [20]. HF is also becoming a model organism for studying interactions between galling insects and host plants [21]. The insect has an extraordinary ability to manipulate wheat development. A single larva is sufficient to induce formation of nutritive cells at the feeding site, to inhibit wheat growth [22], and to reprogram physiological pathways of infested plants [23], [24]. Much of the host manipulation by HF is likely achieved through salivary secretions [25]–[27] but a role of HF-associated bacteria in plant manipulation cannot be excluded. Bacteria have been found both in HF and HF-infested wheat [28], [29], but no systematic survey has been conducted. The objectives of this study were to determine if HF-associated bacteria were transmitted maternally, if bacteria were essential for the insect to survive on wheat, and to survey the composition of bacteria associated with HF and HF-infested wheat.

## Materials and Methods

### Insects

The insect population was derived from a field infestation collected from Ellis County, Kansas [30], [31].

### Culturing bacteria from HF and HF-infested wheat

Insects were surface-sterilized as described by Howard et al. [32]. Surface-sterilized insects were placed into an Eppendorf tube with sterile water and homogenized. The homogenate was spread onto NA plates and incubated aerobically at either 20°C or 37°C for 24 to 120 h to isolate bacterial colonies with different growth rates. Colonies obtained were then individually streaked onto fresh NA plates for re-purification. Liquid cultures of pure colonies were stored in 30% glycerol solutions at −80°C. To access unculturable bacteria, PCR was performed using a pair of primers that are universal for the bacterial 16 S rRNA gene and HF total larval DNA as template. The resulting PCR products were sequenced directly.

To isolate bacterial colonies from HF-infested wheat, wheat tissues at the feeding site eight days post infestation (DPI) were collected after the removal of HF larvae. The collected wheat tissues were homogenized, and individual bacterial colonies were isolated as described in isolation of bacterial colonies from HF individuals.

### Determination of colony forming units

For colony forming units (CFUs) determination, surface-sterilized insects were individually homogenized in 300 µl of sterile water. A five µl aliquot from each homogenate was diluted 10-fold with a total of four serial dilutions. A 50 µl sample of each diluted homogenate was spread on separate NA plates. Bacterial growth was examined 24–36 h later. An average count of bacterial colonies was taken from three replicates.

### DNA extraction, amplification, and sequencing

Individual bacterial colonies were grown at 37°C in separate tubes containing liquid Luria broth (LB) media overnight. DNA was extracted using a cetyl trimethylammonium bromide (CTAB) method [33]. The 16S DNA from individual bacteria was PCR-amplified using the universal primer pair 27F and 1492R (Table S1). PCR reactions were performed in 25 µl of a solution containing 10 ng of bacterial DNA, 1× PCR mix (Promega, Madison, WI) and 0.32 mM of each primer. PCR was performed with an initial 5-min denaturation at 95°C, followed by 30 cycles of 30 sec at 94°C, 30 sec at 55°C, and 30 sec at 72°C, and a final 5-min extension at 72°C. PCR products were purified using a QIAquick Kit (Qiagen, Valencia, CA) and sequenced using a single PCR primer at the KSU sequencing center.

### Identification of bacteria through culture-independent approach

To identify bacteria without culturing, the 16S DNA was amplified through PCR using the universal primers 27F and 1492R (Table S1) with total DNA from whole insects as template. PCR products were run on a 1.2% agarose gel. The expected DNA band was cut out from the gel and purified using the QIAquick Kit. PCR fragments were cloned into pGEM®-T (Promega). Individual plasmid DNA samples were extracted and were sequenced. DNA sequencing was carried our using M13 forward and reverse universal primers on an ABI Prism 3700 DNA Analyzer at Kansas State University sequencing facility.

### Visualization of bacteria in eggs through florescent *in situ* hybridization

To determine if bacteria are transmitted transovarially, we used fluorescent *in situ* hybridization (FISH). Fresh eggs were fixed in 4% paraformaldehyde (PFA, pH 7) in phosphate buffered saline (PBS, 137 mM NaCl, 2.7 mM KCl, 4.3 mM Na_2_HPO_4_, 1.47 mM KH_2_PO_4_, pH 7.4) for 3 h at room temperature (RT). To increase permeability, eggs were dehydrated in an ethanol series (2×30 min in each 70% and 96% ethanol then 2×20 min in 100%) and then washed with PBS. Eggs were then treated with proteinase K (50 µg/ml in PBS) for 15 min, followed by a PBS wash. To quench any auto-fluorescence, eggs were treated with 6% H_2_O_2_ in ethanol overnight [34]. Oligo EUB338 (Table S1) was labeled with Alexa Fluor-488 and used as probe. Hybridization was performed at 46°C for 3 h in a solution containing 900 mM NaCl, 20 mM Tris/HCl, 35% formamide, 0.01% SDS, and 5 ng/µl of the probe. Eggs treated with RNAase served as negative controls. After hybridization, eggs were washed twice with a buffer containing 80 mM NaCl, 20 mM Tris/HCl (pH 8.0), 5 mM EDTA, and 0.01% SDS at 46°C for 30 min. The eggs were then counterstained with propidium iodide and mounted in a glass slide, and were imaged on a Zeiss LSM 5 PASCAL (laser scanning confocal microscope) at KSU Microscopy Facility. This instrument provided three dimensional reconstructions from egg different sections.

### Detection of specific bacterial groups in eggs through PCR

PCR was used to detect the presence of specific bacterial genera in HF eggs. PCR primers were designed to be genera-specific based on published sequences (Table S1) or sequences obtained in the present study. PCR reactions were performed using 100 ng of HF egg DNA as template as described above.

### Antibiotic treatments of plants

Since there is no HF artificial diet, antibiotics were applied directly to wheat seedlings. Ten to 15 wheat seedlings (Karl92) were grown in individual pots. The plants in a mesh cage were infested at the 1.5-leaf stage with one HF female per plant. Antibiotics used for treatments were kanamycin (10 mg/ml), penicillin (5 mg/ml), rifampicin (1 mg/ml), ampicillin (5 mg/ml), streptomycin (5 mg/ml), gentamicin (1 mg/ml), and a mixture of kanamycin and streptomycin (10 and 5 mg/ml). The antibiotics (20 ml solution per pot per day) were sprayed onto wheat seedlings once a day starting from four DPI for a total of four consecutive days. Control plants were sprayed with water. Each treatment was repeated twice and each replication had 15 to 20 plants. Live insects were counted in infested plants 23 DPI.

To determine if larval hatch and migration to the feeding site were affected, the kanamycin/streptomycin mixture was applied when eggs had just been laid. The antibiotics were again applied once per day for four consecutive days. Before spraying, eggs on a plant were counted and the plant was tagged. After another four days, larvae at the feeding site were counted, and the successful hatch/migration rate was calculated.

### Antibiotic treatments of HF larvae

To examine if there was any direct, acute toxicity of antibiotics on HF larvae, fresh HF larvae were soaked directly in a kanamycin/streptomycin solution for 24, 48 and 72 hrs. Larvae soaked in water were taken as controls. Individual larva was then placed onto the leaf axel of wheat seedlings with one insect per plant. These larvae were able to enter into the plant and establish a feeding site. Live larvae were recorded 18 days after the larval placement on wheat leaves.

### Quantitative real-time PCR

Quantitative real-time PCR (qPCR) were carried out with iQ SYBR green super mix using primers that targeted specific bacterial groups (Table S1) as described previously [35]. Each reaction was carried out with one µg total DNA from whole insects as template, 0.5 µM each primer and 12.5 µl iQ SYBR green super mix in 25 µl total volume. Three biological replications were performed. Quantification of 16S DNA in HF was calculated by subtracting cycle threshold (Ct) values from the corresponding actin Ct values. The relative abundance of 16S DNA was determined by the expression 2^−ΔCt^.

### Statistical analysis

Significance of differences between treatments and controls was analyzed using an F-test in SAS. Differences between two treatments were analyzed using Tukey's HSD. Insect survival and mortality data with different antibiotic treatments were analyzed using ANOVA.

## Results

### Adult female hind gut is full of bacteria and ends at the ovarioles

**Figure 1:**
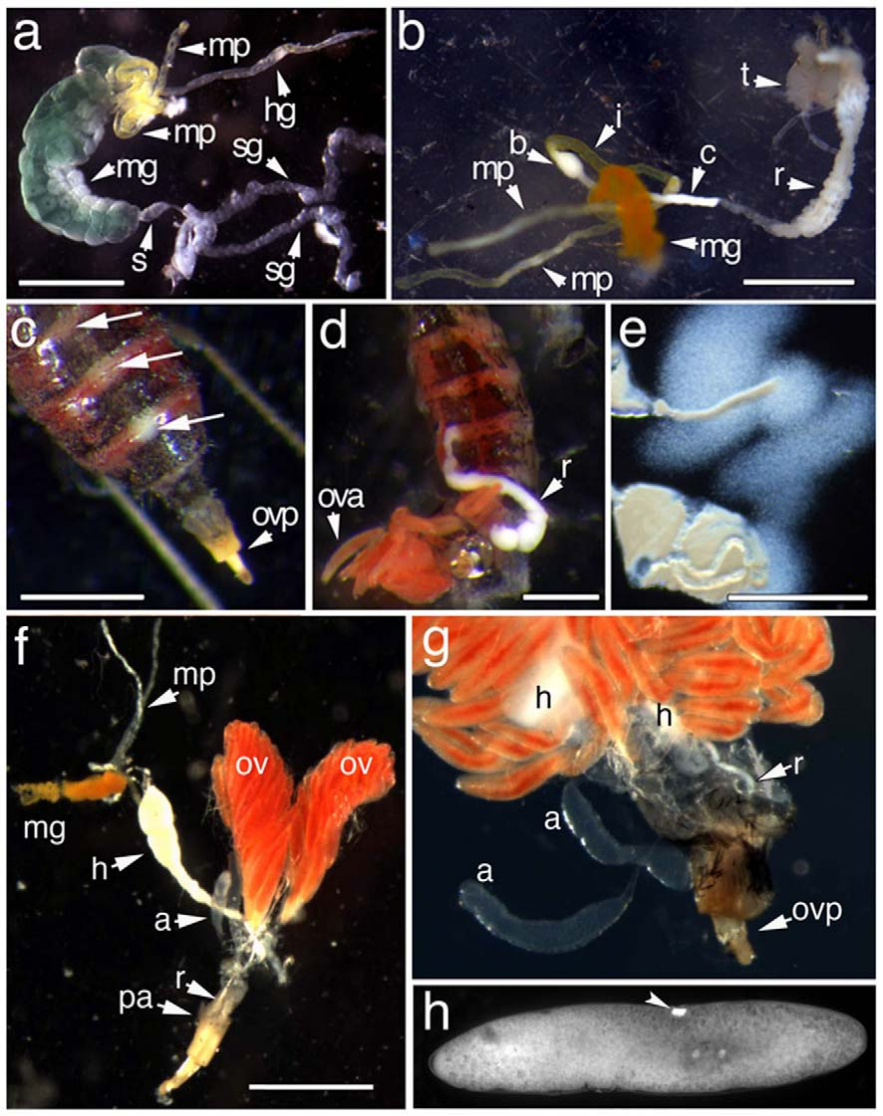
The Hessian fly gut supports the maternal transmission of microflora into eggs. **Panel a:** The alimentary system of a third-instar Hessian fly larva (a non-feeding stage) is composed of paired salivary glands (sg), a short stomatodeum (s), midgut (mg), paired yellow malpighian tubules (mp), and narrow hindgut (hg). **Panel b:** The alimentary system of an adult Hessian fly male is composed of the midgut (mg), paired malpighian tubules (mp), and a hindgut divided into an ileum (i), colon (c), and rectum (r). A bulb (b) dividing the ileum and the colon is visible, as are the testes (t). **Panel c:** The bacteria-laden rectum is visible as a white mass below the dorsal intersegments of the adult female abdomen (long arrows); ovp = ovipositor. **Panel d:** The rectum (r) and a few ova (ova) are visible after making a lateral opening in the female's abdomen. **Panel e:** Laceration of the rectum releases millions of bacteria into the dissection medium (Ringer's solution). **Panel f:** Dissection displaying the adult female midgut (mg), malpighian tubules (mp), hindgut (h), ovaries (ov), and accessory glands (a). The rectum (r) terminates above the postabdomen (ovipositor, pa) with the common oviduct and the accessory glands. **Panel g:** Dissection showing the terminus of the rectum (r), the accessory glands (a), and the ovipositor (ovp). **Panel h:** DAPI-stained binucleate zygote showing the positive-staining bacteriocyte-like structure present in each fertilized egg (arrowhead).

The internal morphology of the HF female abdomen clearly indicated that anatomical modifications have evolved to both propagate bacteria in the hindgut and transmit those bacteria to HF ovarioles. The alimentary system of non-feeding third-instar female larvae is composed of a short proctodeum, a relatively large midgut (mesenteron), two yellow malpighian tubules, and a relatively short and narrow hindgut (proctodeum, Fig. 1a). During pupal development the contents of the midgut change in color, from green to red, and the hindgut lengthens and develops. By the time the adult emerges, the hindgut is clearly composed of an ileum, a colon, and a rectum (Fig. 1). In adult males, the alimentary system is very different from that of a female. Specifically, the colon is a narrow, yellow, transparent tube that leads to a small white bulb that demarcates the end of the ileum and the beginning of the colon. The colon is a narrow white tube about twice the length of the ileum that leads to the rectum (Fig. 1b). In adult females, the hindgut is visible below the posterior dorsal intersegments of the abdomen (Fig. 1c), and filled with bacteria (Fig. 1c–g). In comparison, the relatively underdeveloped male hindgut is not visible and contains no bacteria (Fig. 1b and data not shown). The posterior of the hindgut gradually narrows to form a long thin rectal tube that terminates against the internal dorsal surface of the eighth abdominal segment, above the postabdomen (Fig. 1g). It appears that the gut of the adult female allows bacterial fermentation and then delivers bacteria to the ova as they enter the ovipositor. The female hindgut could also serve as a nutrient delivery system.

### Bacteria in different developmental stages of HF

**Figure 2:**
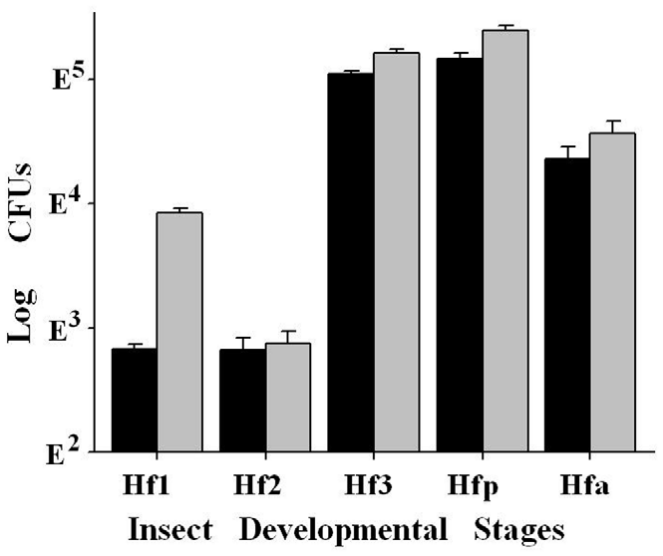
Colony forming units (CFUs) of bacteria in HF first (Hf1), second (Hf2), and third (Hf3) instars, pupae (Hfp), and adults (Hfa). Black bars represent CFUs per larva while gray bars represent CFUs per mg of insects.

To estimate the number of culturable bacteria in different developmental stages, a CFU assay was conducted (Figure 2) on whole insects. HF pupae (1.5×10^5^ per insect) and the third, non-feeding instar larvae (1.1×10^5^ per insect) had the highest CFUs, followed by adults (2.3×10^4^ per insect). First (6.8×10^2^ per insect) and second (6.7×10^2^ per insect) instars exhibited much lower CFUs.

### Maternal transmission of HF-associated bacteria

**Figure 3:**
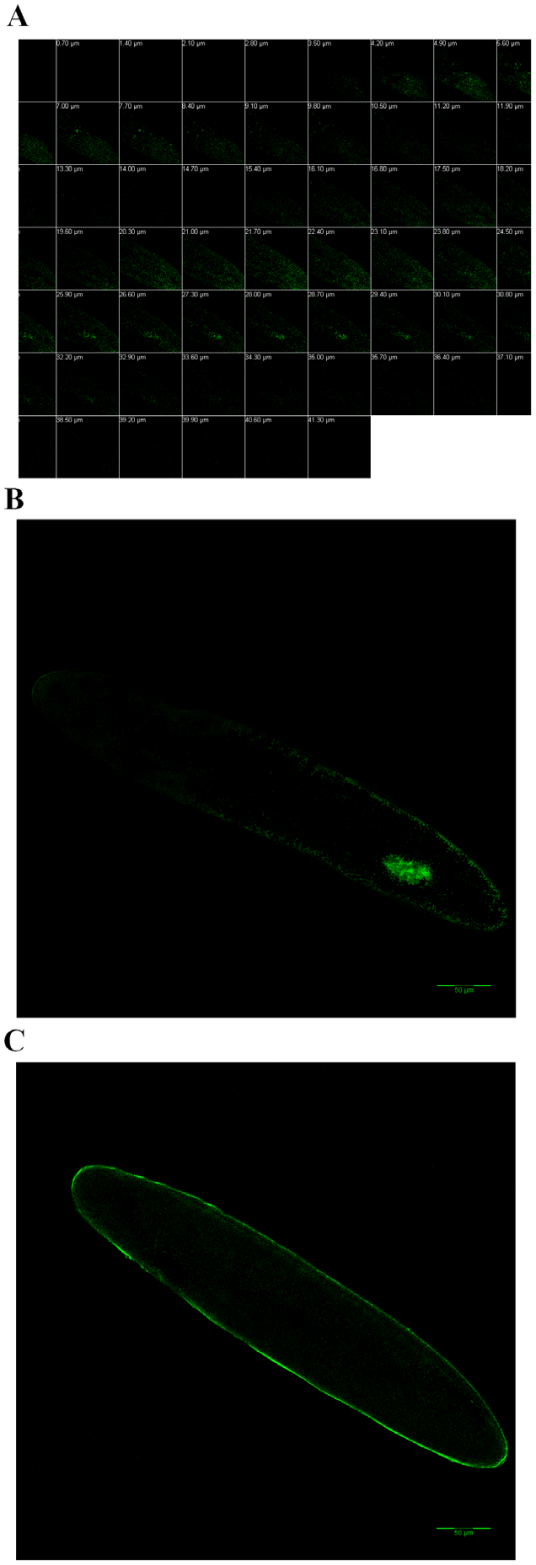
Whole-mount FISH of bacteria with the EUB338 probe in the HF egg. **Panel A:** Different optical sections of hybridized egg at various depths (shown on top left corner for each) from the surface. **Panel B:** An enlarged image of the optical section at 27.30 µm depth. The white arrow points to the specific signal region. **Panel C:** An enlarged image of the optical section at 27.30 µm depth from the RNAase-treated egg.

The bacterial locale specificity in the female hindgut suggested that some of the HF-associated bacteria were likely transmitted maternally to the next generation. To determine if that is the case, FISH with a universal 16S rRNA probe was performed in HF eggs. Under a laser scanning confocal microscope, we were able to see egg sections through optical sectioning (Figure 3A). A hybridization signal was detected near the apical portion of an egg at 25.90 µm below the surface. The specific signal was visible up to 30.10 µm depth, but not thereafter. A closer observation at the 27.30 µm section revealed that several irregular signal spots were located next to each other and formed a rough oval shape (Figure 3B), which was likely a bacteriocyte [36]. No specific signal observed in control RNAase-treated egg preparations (Figure 3C).

**Figure 4:**
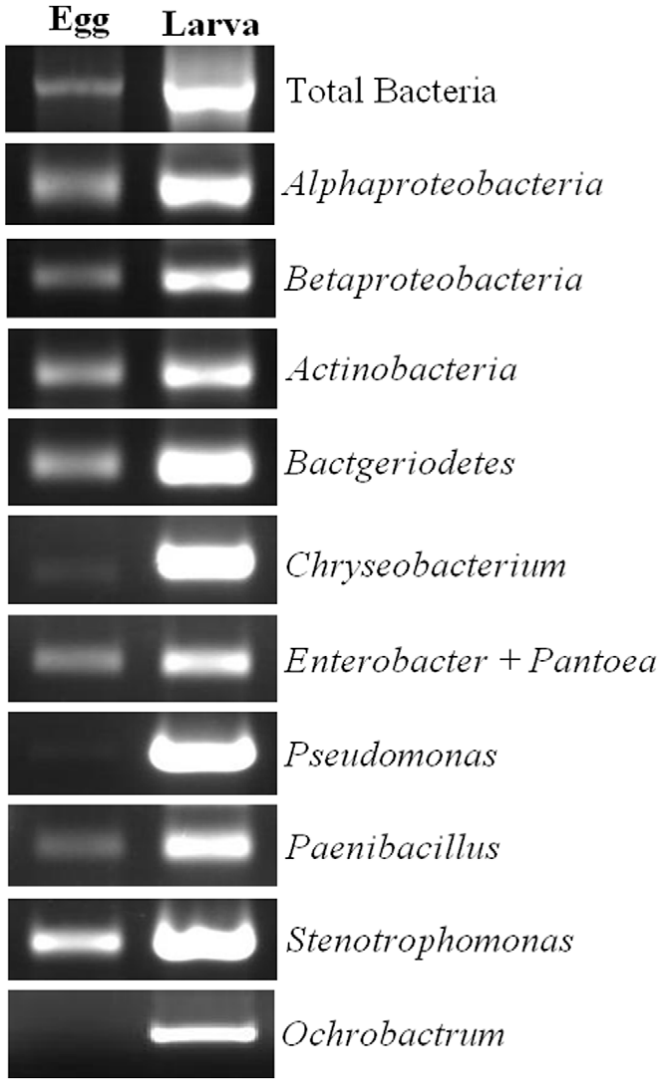
PCR detection of different bacteria in eggs and larvae of HF. For total bacterial detection, the universal primer pair 27F and 1492R were used. All the primers are given in the Table S1.

To further identify different types of bacteria that were present in HF eggs, PCR reactions with primers specific to different bacterial classes or genera were carried out. Primers specific to bacterial classes *Alphaproteobacteria*, *Betaproteobacteria*, *Actinobacteria*, and *Bacteriodetes* all detected signals (Figure 4). Primers specific to bacterial genera *Enterobacter/Pantoea*, *Paenibacillus*, and *Stenotrophomonas* detected relatively strong signals. Primers specific to bacterial genera *Chryseobacterium* and *Pseudomonas* detected weak signals. Primers specific to bacterial genus *Ochrobactrum* detected no signal.

### Impact of antibiotics on HF larval survival

**Figure 5:**
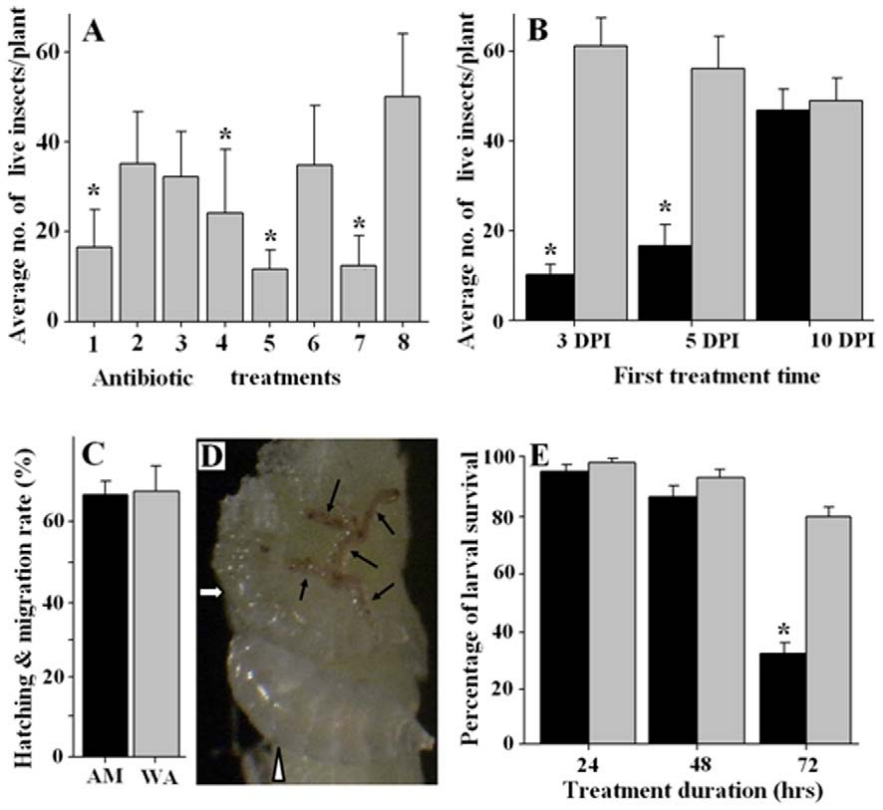
Impact of antibiotics on HF larval survival. The numbers of insects that survived on wheat seedlings after an antibiotic treatment were determined 24 DPI. The numbers of larvae that survived and passed into the pupal stage were expressed as mean±S.E. per plant with a 95% confidence interval. In panels B, C, and E, black bars represent antibiotic treatment whereas gray bars represent water-treated controls. An asterisk (*) represents a statistically significant (*P*≤0.5) difference between treatment and control. **Panel A:** Average number of HF larvae that survived on wheat seedlings treated with different antibiotics. The numbers 1 to 8 represent treatment with kanamycin, penicillin, rifampicin, ampicillin, streptomycin, gentamicin, a mixture of kanamycin and streptomycin, and water, respectively. **Panel B:** Effective time for antibiotic treatment. Wheat seedlings were initially treated with the mixture of kanamycin and streptomycin on day 3, 5, and 10 DPI, respectively. After the initial treatment, the antibiotic mixture was applied once per day for four consecutive days. **Panel C:** Lack of impact of the kanamycin/streptomycin mixture on larval hatching and migration into the feeding site. The hatching and migration rate was calculated from a total of 587 eggs in antibiotic-sprayed plants and 384 eggs in water sprayed plants. Eggs were counted 48 hrs after infestation. Larvae that successfully migrated to the base of the plants were counted 7 DPI. The differences in percent hatching and migration rate were compared by fisher's probability test (*P* = 0.216). **Panel D:** Dead larvae on wheat seedlings treated with the kanamycin/streptomycin mixture. Each black arrow points to a dead first instar larva. The white arrow points to a dead, deformed second instar larva. The white triangle points to a live, second instar larva. **Panel E:** Impact of the kanamycin/streptomycin mixture on larval survival by direct larval soaking. Total numbers of larvae tested were 117, 115, and 218 for 24, 48, and 72 hrs antibiotic exposure, respectively; and 113, 119, and 220 for 24, 48, and 72 hrs water exposure. The numbers of live insects were counted at 24 days after the treatments.

To determine the impact of bacteria on HF larval survival, we used different antibiotics to deprive bacteria of the insect. In general, antibiotic treatments had a negative impact on HF larval survival (Figure 5A). The survival rates of HF larvae were 33%, 70%, 64%, 48%, 23%, 69%, and 25% after treatment with kanamycin, penicillin, rifampicin, ampicillin, streptomycin, gentamicin, and a kanamycin-streptomycin mixture respectively.

Since kanamycin and streptomycin were the most effective antibiotics, a more detailed study was carried out with a mixture of these two antibiotics. To determine the effective time for antibiotic treatments, the kanamycin/streptomycin mixture was applied to wheat seedlings at three different time intervals: 3–7, 5–9, and 10–14 days after HF adult infestation. The numbers of larvae that survived on plants treated at the 3–7, and 5–9 day intervals was 3–5 times less than that on control plants (F_1,78_ = 238.37 and *P*<0.0001 for the 3 to 7 interval, and F_1,78_ = 85.84 and *P*<0.0001 for the 5 to 9 interval) (Figure 5B). No difference was found in insect survival between plants treated at the 10–14 day interval and control plants (F_1,78_ = 0.41, *P* = 0.5241).

To exclude other possible ways that could result in reduction of insects in antibiotic-treated plants, we examined the effect of antibiotic treatments on the rates of egg hatch and successful larval migration into the feeding site (Figure 5C). No difference was observed between antibiotic-treated plants and water-treated controls in terms of egg hatch and larval migration (F_1,22_ = 1.62, *P* = 0.216). On the other hand, numerous larvae were found dead at the feeding site of antibiotic-treated seedlings (Figure 5D). Dead larvae were rarely found at the feeding site of control plants [37]. The impact of the kanamycin/streptomycin mixture on larval survival was also examined by directly soaking larvae in the antibiotic solution, and then individually placing the larvae back onto wheat seedlings. There was a small decrease (*P*>0.05) in survival after the antibiotic soak for 24 and 48 hrs, but a dramatic decrease (*P*<0.0001) after soak for 72 hrs (Figure 5E).

### Culturable bacteria from different developmental HF stages

To identify major culturable bacteria associated with HF, a total of 482 bacterial colonies were isolated from different stages of the insect. Of these colonies, 284 were randomly chosen to sequence their 16S rRNA genes. A total of 270 high quality sequences were obtained, with 87, 65, 37, 49, and 32 derived from bacterial colonies isolated from first, second, and third instars, pupae, and adults, respectively.

Bacteria from four phyla were identified, including *Proteobacteria*, *Firmicutes*, *Actinobacteria*, and *Bacteroidetes* (Table S2). The composition of bacteria associated with the first and second instars was very similar at the levels of phyla and class. At the level of genus, however, there were differences in bacterial compositions between first and second instars. *Proteobacteria* was still predominant in third instar larvae and pupae, but its proportion reduced. The decrease in relative proportion of *Proteobacteria* was due to an increase in *Firmicutes* and *Bacteriodetes* in third instar larvae; and an increase in *Actinobacteria* and *Bacteriodetes* in pupae.

The bacterial composition obtained from HF adults was quite unique. The most abundant bacteria isolated from adults belonged to phyla *Firmicutes*, representing 75.0% of total bacteria. The proportion of *Proteobacteria* reduced to only 12.4%. *Bacillus* (62.5%), *Staphylococcus* (12.5%), *Sphingobacterium* (6.3%), *Enterobacter* (3.1%), *Stenotrophomonas* (3.1%), and *Arthrobacter* (3.1%) were major genera in adults.

### Culturable bacteria from HF-infested susceptible wheat

A total of 149 bacterial colonies were isolated from HF-infested wheat. Thirty-nine 16S rRNA sequences were obtained. The bacteria from infested wheat also belonged to the four main phyla cultured from HF, namely 51.3% *Proteobacteria*, 33.3% *Firmicutes*, 7.7% *Bacteroidetes*, and 5.1% *Actinobacteria* (Table S2). For *Proteobacteria*, the *Gammaproteobacteria* were again the most abundant (35.9%), followed by *Betaproteobacteria* (12.8%) and *Alphaproteobacteria* (2.6%). *Enterobacter* (23.1%), *Bacillus* (23.1%), *Paenibacillus* (7.7%), *Chryseobacterium* (7.7%), *Achromobacter* (6.1%), *Arthrobacter* (5.1%), *Pantoea* (2.6%), *Pseudomonas* (2.6%), *Staphylococcus* (2.6%), and *Microbacterium* (2.6%) were the major genera in HF-infested susceptible wheat. No bacterial colonies could be obtained from non-infested wheat.

### Major bacteria in HF identified through a culture-independent approach

Since culturing could only identify bacteria culturable under our conditions, a culture-independent, PCR-based method was also used for a more comprehensive analysis. For this approach, we analyzed bacterial compositions of first instars, pupae, and adults. A total of 233 high quality 16 rRNA sequences were obtained from 316 clones. Among them, 154, 59, and 20 sequences were derived from first instars, pupae, and adults, respectively. The bacteria identified from the culture-independent analysis included the four phyla identified from the culturing analysis described previously. In addition, a new phylum, *Aquificae*, was also detected from the culture-independent analysis. The relative abundance of *Proteobacteria*, *Firmicutes*, *Actinobacteria*, *Bacteroidetes*, and *Aquificae* was 75.8, 4.6, 14.4, 2.6, and 2.6%, respectively, in first instars; 91.5, 3.4, 3.4, 0, and 0% in pupae; and 65.0, 0, 20, 15.0, and 0% in adults (Table S2).

At the genus level, *Acinetobacter* (53.6%), *Ochrobactrum* (6.5%), *Alcaligenes* (5.2%), *Kocuria* (5.2%), *Nitrosomonas* (3.9%), *Bacillus* (3.9%), *Arthrobacter* (3.9%), *Arcanobacterium* (3.3%), and *Sphingobacterium* (2.6%) were the major genera in first instars. *Pseudomonas* (25.4%), *Acinetobacter* (18.6%), *Klebsiella* (18.6%), *Enterobacter* (15.3%), *Stenotrophomonas* (3.4%), *Paenibacillus* (3.4%), and *Microbacterium* (3.4%), were major genera in pupae. *Ochrobactrum* (55.5%), *Alcaligenes* (10%), *Arthrobacter* (10.0%), *Microbacterium* (5.0%), and *Sphingobacterium* (5.0%) were major genera in adults.

### Impact of antibiotics on bacterial abundance in HF

**Figure 6:**
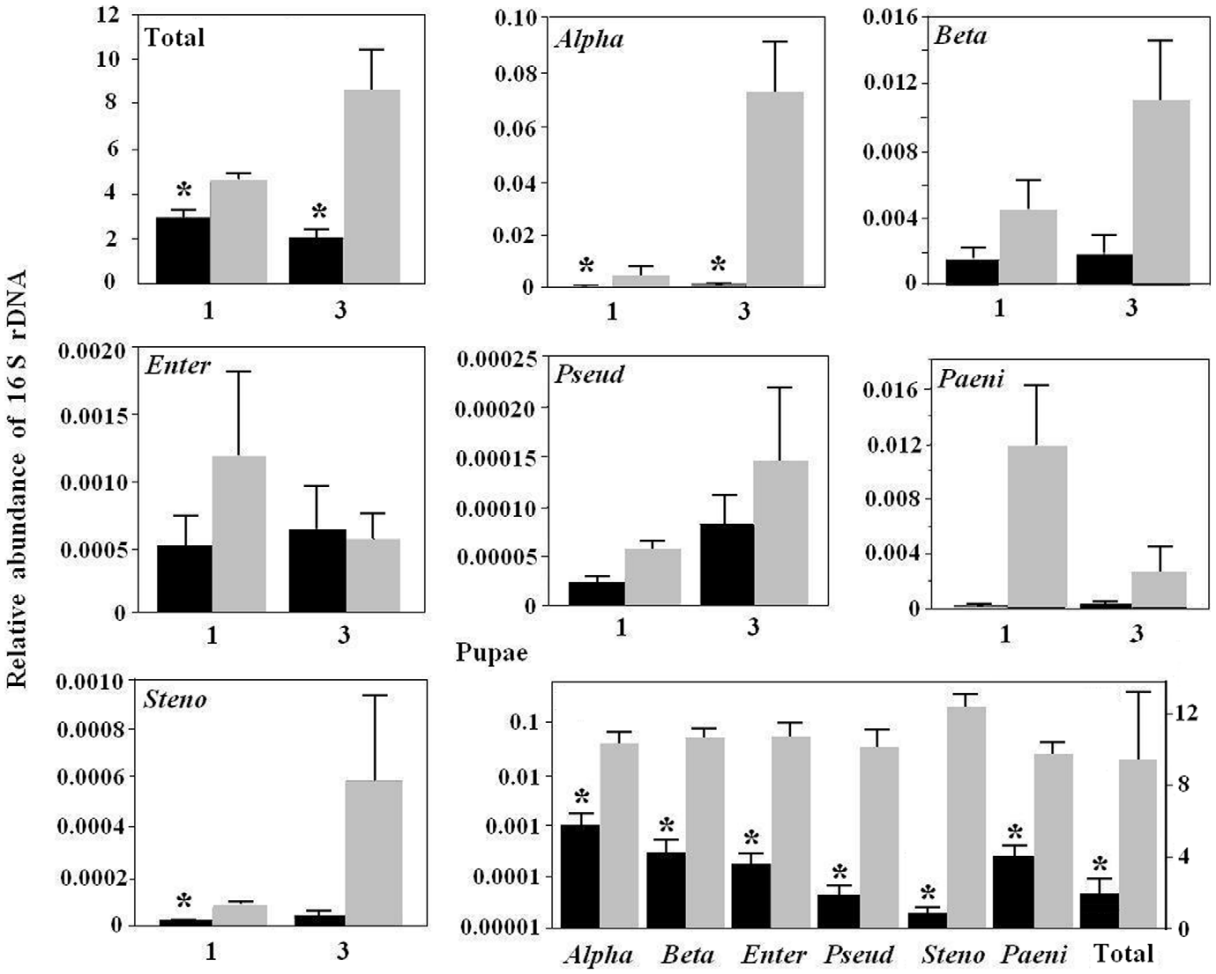
Relative abundance of the 16S DNA of different bacteria associated with HF following antibiotic- (black bars) and water (gray bars) - treatments. A mixture of kanamycin and streptomycin was used for antibiotic treatment. The relative abundance of total and specific groups of bacteria was determined by qPCR using universal and group-specific primer pairs listed in Table S1. The mean abundance (± S.E.) was derived from three biological replicates. An asterisk (*) represents a statistically significant (*P*≤0.5) difference between treatment and control. *Alpha*, *Beta*, *Enter*, *Pseu*, *Paeni*, *Steno*, and Total represent bacterial groups of classes *Alphaproteobacteria* and *Betaproteobacteria*; and genera *Enterobacter*, *Pseudomonas*, *Paenibacillus*, and *Stenotrophomonas*; and total bacteria; respectively. The numbers “1” and “3” under the abscissae of the first seven panels represent 1- and 3-day old larvae. The last panel represents the relative abundance of different bacterial groups as well as total bacteria in HF pupae. The pupae for the antibiotic treatment were from a small portion of larvae survived the antibiotic treatment. The relative abundance of bacterial groups was shown in log scale on the left ordinate whereas the relative abundance of total bacteria was shown in normal scale on the right ordinate.

The kanamycin-streptomycin mixture reduced the population size of total bacteria associated with HF larvae significantly (Figure 6). In antibiotic-treated plants, total bacterial 16S DNA was 36% (t = 3.024, df = 4, *P*<0.05) and 76% (t = 3.428, df = 4, *P*<0.05) lower in one and three day-old larvae, respectively. For different bacterial groups, *Alphaproteobacteria* was 87% (t = 1.244, df = 4, *P*<0.05) and 99% (t = 3.918, df = 4, *P*<0.05) lower in one and three day-old larvae. The overall trend suggested a reduction in the 16S DNA contents corresponding to bacterial groups *Betaproteobacteria*, *Enterobacter*, *Pseudomonas*, *Paenibacillus*, and *Stenotrophomonas*. However, the degree of reduction was not as dramatic as *Alphaproteobacteria* and variations among replicates were larger.

**Figure 7:**
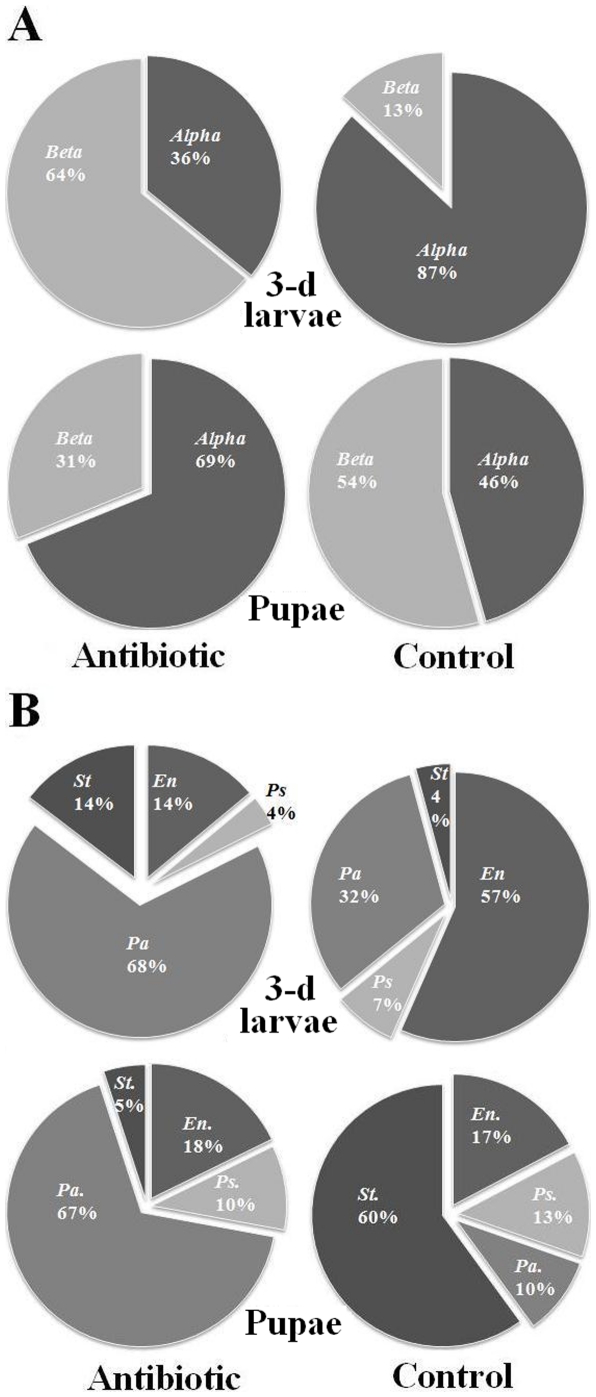
Alternation in bacterial composition after antibiotic treatments. A mixture of kanamycin and streptomycin was used for antibiotic treatment. **Panel A:** Proportions of *Alphaproteobacteria* (*Alpha*) and *Betaproteobacteria* (*Beta*) in antibiotic-treated (Antibiotic) and control 3-day old larvae (3-d larvae) and pupae. **Panel B:** Proportions of bacterial groups *Enterobacter* (*En.*), *Pseudomonas* (*Ps.*), *Paenibacillus* (*Pa.*), and *Stenotrophomonas* (*St.*) in antibiotic-treated and control larvae and pupae.

Irrespective of antibiotic treatments, a small proportion of larvae survived and became pupae. The relative abundances of the 16S DNA from total bacteria and from specific bacterial groups in control and antibiotic-surviving pupae were compared (Figure 6). Relative abundance of 16S DNA from total bacteria was 84.0% less in antibiotic-treated pupae. The relative abundance of 16S DNA from specific bacterial groups was significantly reduced for all groups, but the percentage of reduction varied among them. Due to the different impact of antibiotics on different bacterial groups, the bacterial compositions in HF larvae and pupae were shifted following the antibiotic treatment (Figure 7). Under control conditions, the relative proportion of *Alphaproteobacteria* to *Betaproteobacteria* was 87% to 13% in three-day old larvae (Figure 7A). After the antibiotic treatment, the relative proportion of *Alphaproteobacteria* to *Betaproteobacteria* was 36% to 64%, indicating that *Alphaproteobacteria* were more sensitive to antibiotics than *Betaproteobacteria*. On the other hand, in the larvae that survived the antibiotic treatment and became pupae, the proportion of *Alphaproteobacteria* was 69%; significantly higher than the 46% in control insects. Changes in bacterial composition after the antibiotic treatment were also apparent at the genus level (Figure 7B). Primers specific to four genera, three from *Gammaproteobacteria* (*Enterobacter*, *Pseudomonas*, and *Stenotrophomonas*) and one from *Firmicutes* (*Paenibacillus*), again detected dramatic differences in the relative abundance of their DNA between antibiotic-treated and control insects. The percentages for *Enterobacter*, *Pseudomonas*, and *Stenotrophomonas*, and *Paenibacillus* were 57, 7, 4, and 32 in control three-day old larvae; and the percentages changed to 14, 4, 14, and 68 in the same age larvae after the antibiotic treatment. The percentages for *Enterobacter*, *Pseudomonas*, and *Stenotrophomonas*, and *Paenibacillus* were 17, 13, 60, and 10 in control pupae; and the percentages changed to 18, 10, 5, and 67 in the pupae derived from larvae that survived the antibiotic treatment.

## Discussion

In this study, we conducted an analysis on the possible transmission mechanism, essentiality, and composition of bacteria associated with HF. Our results revealed that most HF-associated bacteria were likely to be transmitted maternally through eggs. A bacteriocyte-like structure was observed in developing eggs by FISH (Figure 3B). PCR with primers specific to individual bacterial groups revealed that most of bacteria detected in other stages of HF were also present in eggs (Figure 4). The presence of huge numbers of bacteria in the hindgut of HF female adults specifically (not present in male adults), and the fact that the hindgut ends in ovarioles may provide an unique way among insects to allow bacteria somehow entered into developing eggs from the hindgut. However, the exact mechanism for the physical transfer of HF-associated bacteria remains to be delineated. In aphids, *Buchnera spp.* are liberated from bacteriocytes in adult insects, circulated in body fluids, and then enter fertilized eggs through an opening on the egg surface [35]. In whiteflies, psyllids, mealybugs, and cockroaches, bacteria are transferred into eggs by transferring whole bacteriocytes from female adults directly into eggs [38]. In tsetse flies, bacterial symbionts *Wigglesworthia glossinidia* and *Sodalis glossinidius* are transmitted into next generation through milk glands [39].

The maternal transmission of HF-associated bacteria suggests a close relationship between bacterial symbionts and the insect [40]. In general, the essentiality of symbiotic bacteria for insect survival is often examined by depriving insects of bacteria with diets containing antibiotics. In this study, HF-associated bacteria were targeted with antibiotics, and the resulting effect on HF survival, if any, was determined. Because of the lack of a HF artificial diet, we conducted antibiotic treatments by applying antibiotic solutions onto wheat leaves, assuming that the antibiotics could penetrate into wheat tissues, and eventually entering HF larvae along with other food gradients. As shown in Figure 5A, the application of different antibiotics resulted in 30–77% decrease in HF larval survival rates. The reduction in HF survival was correlated with the deprivation of bacteria from the host insect. Therefore, the loss of bacteria was likely the reason for the reduction in HF larval survival. However, antibiotics, due to their inherent toxicity, could have been responsible for lowering the survival of the insect. Accordingly, we conducted further experiments to see if there was toxicity of antibiotics directly to the insect, and our results suggested that the high mortality of HF larvae was not due to toxicity of antibiotics directly to the insect. First, the application of antibiotics when HF larvae were five days old (during the transition from first to second instar) did not increase larval mortality (Figure 5B, 10 DPI), indicating that the antibiotics did not have a toxic effect on larval metabolism. Second, directly soaking HF larvae in an antibiotic solutions for 24 and 48 hrs resulted in only a small increase in larval mortality (Figure 5E), indicating that the antibiotics did not have an acute, direct toxic effect on HF larvae. The increase in mortality at a later time point was likely due to the depletion of bacteria. In addition, antibiotic treatments have not shown severe direct toxicity to other insects [41]–[44]. Taken together, our evidence suggested that deprivation of bacteria from the insect by antibiotics was the cause for larval mortality and that symbiotic bacteria were essential for larval survival.

The exact roles of bacteria in HF remain to be determined. Symbiotic bacteria perform a range of functions in other insects, including synthesizing essential amino acids [45]–[49], synthesizing vitamins [50], digesting nutrients [47], [51], increasing tolerance of host insects to biotic and abiotic stresses [11], [12], [52]–[54], performing nitrogen fixation [55], [56] and playing a role in insect – plant interactions [13], [15], [16], [57]–[59]. A large number of bacterial species reside in the HF larval gut. These gut bacteria could play a role in synthesis of necessary nutrients for HF larvae, or digestion of nutrients otherwise inaccessible to the insect. Bacteria could also play a critical role in HF – wheat interaction. Approximately 70% of bacterial genera detected in HF larvae through culturing were also found in HF infested wheat (Table S2). Considering the extraordinary ability of HF larvae to manipulate host plants [17], [22] and reprogram metabolic pathways of wheat seedlings [23], [24], it was possible that bacteria were secreted into host plants as part of oral secretions and participated in changing wheat metabolism once they were in wheat seedlings. Further investigation would be needed to examine these possibilities.

It would be interesting to know what types of bacteria were essential for HF larvae to survive. Initial evidence suggested that *Alphaproteobacteria* might be important for HF larval survivability according to their response to antibiotic treatments. *Alphaproteobacteria* was 5.6 time more abundant than *Betaproteobacteria* in 3-day old larvae under control conditions (Figure 6B). After an antibiotic treatment, *Alphaproteobacteria* decreased to almost one third of *Betaproteobacteria* in the same stage of larvae. Associated with the decrease in abundance of *Alphaproteobacteria* and other bacteria was the high death rate of HF larvae. In pupae derived from larvae that survived the antibiotic treatment, *Alphaproteobacteria* were again predominant. These observations suggested that the loss of *Alphaproteobacteria* might be responsible for larval death. Again, further tests are needed to confirm this postulation.

A preliminary survey of the bacterial composition through culturing and culture-independent approaches revealed that diverse bacteria existed in HF and that the bacterial composition changed in different developmental stages of the insect (Table S2). Among the most abundant bacteria detected through culturing were *Enterobacter*, *Pantoea*, *Klebsiella*, *Pseudomonas*, *Stenotrophomonas*, *Staphylococcus*, and *Achromobacter*. *Enterobacter* was the most dominant among cultured bacteria recovered from the three HF larval instars and pupae, with relative abundance ranging from 32–38%. The recovery of *Enterobacter* from all stages of HF indicated a stable relationship between the two partners. Bacterial genera found abundant through the culture-independent method included *Acinetobacter*, *Alcaligenes*, *Nitrosomonas*, and *Ochrobactrum*. *Acinetobacter* was the most dominant, representing 54% of total 16S DNA in the first HF instar. Some bacteria detected through culturing were not detected through the culture-independent approach, suggesting that a more comprehensive survey is needed to identify more HF-associated bacteria.

In summary, diverse bacteria were associated with different developmental stages of HF. Most of these bacteria were transmitted maternally to the next generation indicating an close relationship between the insect and its associated bacteria. Depletion of microbes from HF larvae with antibiotics caused a high mortality of the insect in wheat seedlings, indicating that antibiotic-sensitive microbes were essential for the insect to survive on wheat seedlings. This research is the first systematic analysis of bacteria associated with HF and provides a foundation for future research to elucidate the complex tritrophic interactions among wheat, HF, and their symbiotic bacteria.

## Supporting Information

Table S1 Primers used in this study.
(DOC)

Table S2 Relative frequency (%) of different bacteria derived from different stages of Hessian fly and infested wheat by culture dependent and culture-independent methods.
(DOC)
