# Supplementary material for: Hessian Fly-Associated Bacteria: Transmission, Essentiality, and Composition
Source: PLoS One. 2011 Aug 16;6(8):e23170. doi: 10.1371/journal.pone.0023170 (PMC3156707; doi:10.1371/journal.pone.0023170)
Supplement: Table S1 — Primers used in this study. (DOC) [file pone.0023170.s001.doc]

**Table S1. Primers used in this study***

| **Target group** | **Primer set** | **Primer Sequence (5` to 3`)** | **Reference** | **AT (ºC)** | | **AL (bp)** |
| --- | --- | --- | --- | --- | --- | --- |
| All bacteria | Eub338  Eub518 | ACTCCTACGGGAGGCAGCAG  ATTACCGCGGCTGCTGG | Lane 1991  Muyzer et al 1993 | 53 | | 200 |
| All bacteria | 27F  1492R | AGAGTTTGATCMTGGCTCAG  GGYTACCTTGTTACGACTT | Lane 1991  Lane 1991 | 55 | | 1502 |
| *Alphaproteobacteria* | Eub338  Alf685 | ACTCCTACGGGAGGCAGCAG  TCTACGRATTTCACCYCTAC | Lane 1991  Lane 1991 | 55 | | 365 |
| *Betaproteobacteria* | Eub338  Bet680 | ACTCCTACGGGAGGCAGCAG  TCACTGCTACACGYG | Lane 1991  Overmann et al 1990 | 55 | | 360 |
| *Actinobacteria* | Actino235  Eub518 | CGCGGCCTATCAGCTTGTTG  ATTACCGCGGCTGCTGG | Stach et al. 2003  Muyzer et al. 1993 | 55 | | 300 |
| *Bacteriodetes* | Cfb319  Eub518 | GTACTGAGACACGGACCA  ATTACCGCGGCTGCTGG | Manz et al. 1996  Muyzer et al. 1993 | 60 | | 220 |
| *Chryseobacterium* | 2F  2R | GAGKTCTTTCGGGATCTTGAG  GCTWTCYACACGTRGASAGGT | This study  This study | 55 | | 398 |
| *Enterobacter*  */Pantoea* | 11F  11R | TAGCACAGAGAGCTTGCTCTC  CTGCGGTTATTAACCACAATGCC | This study  This study | 52 | | 404 |
| *Pseudomonas* | 16F  16R | TAGAGAGRWGCWYGCTTCTCTTGA  CAATTACGTATTAGGTAACTGCCC | This study  This study | 65 | | 401 |
| *Paenibacillus* | 18F  18R | AAGAGAACTGGAAAGACGGAGC  AGCAGTTACTCTCCCAAGCG | This study  This study | 52 | | 283 |
| *Stenotrophomonas* | 20F  20R | CAGCACAGGAGAGCTTGCTCT  AACCAGGTATTAGCCGGCTGGAT | This study  This study | 55 | | 411 |
| *Ochrobactrum* | 21.22F  21.22R | CAGGATACATAAAATGCCCTGG  TCATTATCTTCACCGGTGAAAGAG | This study  This study | 55 | 286 | |

*AT – Annealing temperature. AL – Amplicon length.
