# Supplementary material for: Hessian Fly-Associated Bacteria: Transmission, Essentiality, and Composition
Source: PLoS One. 2011 Aug 16;6(8):e23170. doi: 10.1371/journal.pone.0023170 (PMC3156707; doi:10.1371/journal.pone.0023170)
Supplement: Table S2 — Relative frequency (%) of different bacteria derived from different stages of Hessian fly and infested wheat by culture dependent and culture-independent methods. (DOC) [file pone.0023170.s002.doc]

**Table S2. Relative frequency (%) of different bacteria derived from different stages of Hessian fly and infested wheat by culture dependent and culture-independent methods.**

|  |  | |  | | Culture-dependent method | | | | | | Culture-independent method | | |
| --- | --- | --- | --- | --- | --- | --- | --- | --- | --- | --- | --- | --- | --- |
| Phyla | | Class | | Genera | Hf1 | Hf2 | Hf3 | Hfp | Hfa | Wheat | Hf1 | Hfp | Hfa |
| Proteobacteria | | | |  | 90.8 | 90.8 | 62.2 | 73.4 | 12.4 | 51.3 | 75.8 | 91.5 | 65.0 |
|  | | Alphaproteobacteria | | | ND | 1.5 | ND | ND | 3.1 | 2.6 | 8.5 | 5.1 | 55.0 |
|  | |  | | Ochrobactrum | ND | ND | ND | ND | ND | ND | 6.5 | 1.7 | 55.0 |
|  | |  | | Others | ND | 1.5 | ND | ND | 3.1 | 2.6 | 2.0 | 3.4 | ND |
|  | | Betaproteobacteria | | | 1.1 | ND | 2.7 | 6.1 | ND | 12.8 | 9.8 | 3.4 | 10.0 |
|  | |  | | Achromobacter | 1.1 | ND | 2.7 | 6.1 | ND | 5.1 | ND | 1.7 | ND |
|  | |  | | Alcaligenes | ND | ND | ND | ND | ND | ND | 5.2 | ND | 10.0 |
|  | |  | | Nitrosomonas | ND | ND | ND | ND | ND | ND | 3.9 | ND | ND |
|  | |  | | Others | ND | ND | ND | ND | ND | 7.7 | 0.7 | 1.7 | ND |
|  | |  | |  |  |  |  |  |  |  |  |  |  |
|  | | Gammaproteobacteria | | | 89.7 | 89.2 | 59.5 | 67.3 | 9.3 | 35.9 | 57.5 | 83.1 | ND |
|  | |  | | Enterobacter | 37.9 | 35.4 | 29.7 | 32.7 | 3.1 | 23.1 | ND | 15.3 | ND |
|  | |  | | Pantoea | 34.5 | 6.2 | 5.4 | 20.4 | ND | 2.6 | ND | ND | ND |
|  | |  | | Stenotrophomonas | 1.2 | 23.1 | 5.4 | 4.1 | 3.1 | ND | ND | 3.4 | ND |
|  | |  | | Pseudomonas | 2.3 | 9.2 | 13.5 | 6.1 | ND | 2.6 | 1.3 | 25.4 | ND |
|  | |  | | Klebsiella | 4.6 | 9.2 | 2.7 | ND | ND | ND | 1.3 | 18.6 | ND |
|  | |  | | Escherichia | 3.5 | ND | ND | 2.0 | ND | ND | ND | ND | ND |
|  | |  | | Acinetobacter | ND | 4.6 | ND | ND | ND | ND | 53.6 | 18.6 | ND |
|  | |  | | Others | 5.7 | 1.5 | 2.8 | 2.0 | 3.1 | 7.6 | 1.1 | 1.8 | ND |
|  | |  | |  |  |  |  |  |  |  |  |  |  |
| Firmicutes | |  | |  | 6.9 | 7.7 | 29.7 | 4.1 | 75.0 | 33.3 | 4.6 | 5.1 | ND |
|  | | Bacilli | |  | 6.9 | 6.2 | 29.7 | 2.0 | 75.0 | 33.3 | 3.9 | ND | ND |
|  | |  | | Bacillus | 1.2 | 3.1 | 8.1 | 2.0 | 62.5 | 23.1 | 3.9 | ND | ND |
|  | |  | | Staphylococcus | 5.8 | ND | 21.6 | ND | 12.5 | 2.6 | ND | ND | ND |
|  | |  | | Brevibacillus | ND | 3.1 | ND | ND | ND | ND | ND | ND | ND |
|  | |  | | Paenibacillus | ND | ND | ND | ND | ND | 7.7 | ND | 3.4 | ND |
|  | | Not identified yet | |  | ND | 2.5 | ND | 2.1 | ND | ND | 0.7 | 1.7 | ND |
|  | | | |  |  |  |  |  |  |  |  |  |  |
| Actinobacteria | | | |  | 2.3 | 1.5 | ND | 18.4 | 6.3 | 5.1 | 14.4 | 3.4 | 20.0 |
|  | | Actinobacteria | |  | 2.3 | 1.5 | ND | 18.4 | 3.1 | 5.1 | 12.4 | ND | 20.0 |
|  | |  | | Arthrobacter | 1.2 | 1.5 | ND | 18.4 | 3.1 | 5.1 | 3.9 | ND | 10.0 |
|  | |  | | Kocuria | 1.2 | ND | ND | ND | ND | ND | 5.2 | ND | ND |
|  | |  | | Microbacterium | ND | ND | ND | ND | ND | 2.6 | ND | 3.4 | 5.0 |
|  | |  | | Arcanobacterium | ND | ND | ND | ND | ND | ND | 3.3 | ND | ND |
|  | |  | | Others | ND | ND | ND | ND | ND | ND | ND | ND | 5.0 |
|  | | Others | |  | ND | ND | ND | ND | 3.2 | ND | 2.0 | ND | ND |
| Bacteriodetes | | | |  | ND | ND | 10.8 | 4.1 | 6.3 | 7.7 | 2.6 | ND | 15.0 |
|  | | Sphingobacteria | | | ND | ND | ND | 4.1 | 6.3 | ND | 2.6 | ND | 5.0 |
|  | |  | | Sphingobacterium | ND | ND | ND | 4.1 | 6.3 | ND | 2.6 | ND | 5.0 |
|  | | Flavobacteria | |  | ND | ND | 10.8 | ND | ND | 7.7 | ND | ND | 5.0 |
|  | |  | | Chryseobacterium | ND | ND | 10.8 | ND | ND | 7.7 | ND | ND | ND |
|  | |  | | Others | ND | ND | ND | ND | ND | ND | ND | ND | 5.0 |
|  | | Not identified yet | |  | ND | ND | ND | ND | ND | ND | ND | ND | 5.0 |
|  | |  | |  |  |  |  |  |  |  |  |  |  |
| Aquificae | Not identified yet | | |  | ND | ND | ND | ND | ND | ND | 2.6 | ND | ND |

HF1, Hf2, Hf3, Hfp, and Hfa represent first, second, and third instar larvae, pupae, and adults. Wheat represents infested susceptible wheat.

ND represents “not detected”.
